# Supplementary material for: Detecting Sentiment toward Emerging Infectious Diseases on Social Media: A Validity Evaluation of Dictionary-Based Sentiment Analysis
Source: Int J Environ Res Public Health. 2022 Jun 1;19(11):6759. doi: 10.3390/ijerph19116759 (PMC9180278; doi:10.3390/ijerph19116759)
Supplement: Supplementary file 1 [file ijerph-19-06759-s001.zip › ijerph-1726492-supplementary.pdf]

International Journal of
Environmental Research and Public Health

MDPI

Supplemental Material

Detecting Sentiment toward Emerging Infectious Diseases on Social Media: A Validity Evaluation of Dictionary-Based Sentiment Analysis

Sanguk Lee [1], Siyuan Ma [1], Jingbo Meng [1], Jie Zhuang [2] and Tai-Quan Peng [1,*]

[1] Department of Communication, Michigan State University, East Lansing, MI 48824, USA; lswook555@gmail.com (S.L.); masiyua1@msu.edu (S.M.); jingbome@msu.edu (J.M.)
[2] Bob Schieffer College of Communication, Texas Christian University, Fort Worth, TX 76129, USA; jie.zhuang@tcu.edu
* Correspondence: winsonpeng@gmail.com; Tel.: +1-517-355-0221; Fax: +1-517-432-1192

Manuscript Submitted to

International Journal of Environmental Research and Public Health

S1. Article Searching Method

The authors searched articles using three broad categories of keywords; a) sentiment (e.g., emotion, sentiment, mood, and feel), b) computational sentiment analysis (e.g., computational methods, text mining, sentiment analysis, and natural language processing), and c) health issues (e.g., health, disease, infection, and the 37 most searched diseases and conditions provided by CDC). The search procedure returned 486 articles. To filter out irrelevant articles, the authors read the main text of each article and evaluated the relevancy of the article based on the following inclusion criteria: (1) usage of text-based sentiment analysis, (2) analysis of sentiments, and (3) focus on public health issues. Articles meet all the inclusion criteria were retained. Finally, 133 qualified studies were retained. It is notable that a reviewed study could use both DSA and machine learning methods. That is, the sum of the number of DSA articles (n = 85) and machine learning articles (n = 59) is not equal to 133.

S2. Sentiment Classification for DSA

The following technique is used to classify a tweet into negative, positive, neutral, and mixed sentiments. First, using positive and negative scores, we computed the valence and the strength of sentiment. Valence refers to the direction of the sentiment (e.g., positivity versus negativity), whereas the strength of sentiment indicates the intensity of sentiment embedded in a tweet regardless of its positive or negative directionality. We adopted the mathematical definition of valence from Stieglitz and Dang-Xuan (2013)'s study as described in Equation (S1). The strength of sentiment is operationalized following Equation (S2):

$$V_T = |POS_T| - |NEG_T|, \qquad \text{(S1)}$$

$$SS_T = |POS_T| + |NEG_T|, \qquad \text{(S2)}$$

In Equation (S1), V_T is the valence of a tweet. POS_T is the positive score of a tweet, and NEG_T is the negative score of a tweet. The valence of a tweet is computed by subtracting the absolute value of a negative score from the absolute value of the positive

score. In Equation (S2), SS_T is the strength of sentiment in a tweet. The strength of sentiment is computed by summing the absolute value of the positive and negative scores.

Second, a tweet was classified into either negative, positive, neutral, or mixed sentiment based on a topological rule suggested in Table S1. This approach enables us to clearly distinguish neutral sentiment from mixed sentiment. A tweet with neutral sentiment refers to a tweet that has no emotional expression. Following the definition, a tweet that has a 0 score in both valence and strength of sentiment scores was classified as neutral. A tweet represents mixed sentiment when a tweet has the same amount of positive and negative scores. Based on the definition, a tweet that has a 0 score in valence but has a larger value than 0 in the strength of sentiment was classified as a mixed sentiment tweet. Tweets classified as mixed sentiment were excluded as it is not in the interest of the study. A tweet was classified as negative when the valence score of a tweet was negative. A tweet was classified as positive when the valence score of a tweet was positive.

Table S1. Topology of Negative, Neutral, Positive, and Mixed Sentiments.

	**Valence < 0**	**Valence = 0**	**Valence > 0**
Strength of Sentiment = 0	NA	Neutral Sentiment	NA
Strength of Sentiment > 0	Negative Sentiment	Mixed Sentiment	Positive Sentiment

S3. Validity of VADER, SO-CAL, and Support Vector Machine (SVM)

The validity of VADER, SO-CAL, and SVM is evaluated by comparing their sentiment analysis results with manual coding results. Python codes for VADER and SO-CAL were adopted from the GitHub community and modified for the study. Sklearn Python package was used for SVM. For SVM, data were split into the train (n = 6239), validation (n = 1248), and test sets (n = 312) based on the 80/20 split method, which is a common practice for train and test data split in machine learning communities.

Table S2 demonstrates the validation results. SVM outperforms any of DSA. SVM's accuracy (74 %) and Macro Average of F1 (.57) are substantially higher than LIWC's accuracy (57 %) and Macro Average of F1 (.45), which are the highest scores among DSA. Accuracy and Macro Average of F1 of VADER (Accuracy: 38%, Macro Average of F1: .35) and SO-CAL (Accuracy: 47%, Macro Average of F1: .39) are better than SWN (Accuracy: 19%, Macro Average of F1: .18) and adSWN (Accuracy: 21%, Macro Average of F1: .21) and slightly better than ANEW (Accuracy: 33%, Macro Average of F1: .27) and orgSWN (Accuracy: 37%, Macro Average of F1: .30). There is no evidence indicating that VADER and SO-CAL are more valid than LIWC (Accuracy: 57%, Macro Average of F1: .45).

Table S2. Validity Evaluation of VADER, SO-CAL, and SVM in Comparison with Manual Coding Results.

	VADER	SO-CAL	SVM	
	F1	F1	F1	Mean
Neg	0.32	0.31	0.52	0.38
Neu	0.47	0.58	0.84	0.63
Pos	0.25	0.26	0.35	0.36
Macro Average	0.35	0.39	0.57	0.44
Accuracy (%)	38.23	46.78	73.72	52.91
Tweets (*n*)	7769	7799	-	-

Although VADER and SO-CAL may not be more valid than other DSA, as Table S3 demonstrates, problematic textual features used to be associated with the invalidity of other DSA in the main results (See Table 3 in the main text) were resolved when using VADER and SO-CAL. Intensifiers with SO-CAL were the only problematic textual feature still associated with invalidity.

Table S3. The Results of Binary Logistic Regression: Influences of Textual Features on Inconsistency of VADER and SO-CAL.

Textual Features (IVs)	Inconsistency (DV)	
	VADER	SO-CAL
Intercept	-0.41 ***	0.003
Semantic Level		
Embedded hashtags	0.03	0.06
Irrealis	0.13	0.20
Sarcasm	0.48	1.65
Negations	-0.05	0.14
Intensifiers	0.04	0.42 **
Diminishers	-0.18	-0.71
Word-level		
Unconfirmed typos	0.36	-0.09
Lengthened words	0.40	0.22
Irregularly capitalized words	0.08	0.837
Abbreviations	-0.31	0.05
Acronyms	0.10	0.48

Note: * $p < 0.05$, ** $p < 0.01$, *** $p < 0.001$; in DV, consistent condition = 0, inconsistent condition = 1; the number of tweets that include each of the textual features are as follows: embedded hashtags (n = 429), irrealis (n = 429), sarcasm (n = 7), negation (n = 248), intensifiers (n = 260), diminishers (n = 11), unconfirmed typos (n = 35), lengthened words (n = 7), irregularly capitalized words (n = 71), abbreviations (n = 88), acronyms (n = 30); the total sample size is 1969.
